# The waiting room: vector for health education? the general practitioner’s point of view

**DOI:** 10.1186/1756-0500-5-511

**Published:** 2012-09-18

**Authors:** Maxine Gignon, Hadjila Idris, Cecile Manaouil, Oliver Ganry

**Affiliations:** 1 Medical School, Jules Verne University of Picardy, 3 rue des Louvels, F-80000, Amiens, France; 2 Public Health Department, Amiens University Hospital, Amiens, France; 3 Health Education Department, EA3412, UFR-SMBH Leonard de Vinci-Université Paris-Nord, Bobigny, France

**Keywords:** Health education, Health promotion, Family practice, Physician’s office, Health policy, Public health

## Abstract

**Background:**

General practitioners (GPs) play a central role in disseminating information and most health policies are tending to develop this pivotal role of GPs in dissemination of health-related information to the public. The objective of this study was to evaluate use of the waiting room by GPs as a vector for health promotion.

**Results:**

A cross-sectional study was conducted on a representative sample of GPs using semi-structured, face-to-face interviews. A structured grid was used to describe the documents. Quantitative and qualitative analysis was performed. Sixty GPs participated in the study. They stated that a waiting room had to be pleasant, but agreed that it was a useful vector for providing health information. The GPs stated that they distributed documents designed to improve patient care by encouraging screening, providing health education information and addressing delicate subjects more easily. However, some physicians believed that this information can sometimes make patients more anxious. A large number of documents were often available, covering a variety of topics.

**Conclusion:**

General practitioners intentionally use their waiting rooms to disseminate a broad range of health-related information, but without developing a clearly defined strategy. It would be interesting to correlate the topics addressed by waiting room documents with prevention practices introduced during the visit.

## Background

In developed countries, about 75% of a general practitioner’s (GP’s) patients spend time in the GP’s waiting room at least once a year [1-4]. General practitioners are in an unique position to disseminate health information. Most health policies are tending to develop the pivotal role of GPs in disseminating health-related information to the public. In the French health system, GPs are free to distribute prevention materials. These documents may be derived from the French National Institute for Prevention and Health Education or any other organization (e.g. associations, drug companies, or specialist learned societies).

Can waiting time be used to convey health information in primary care? A literature search revealed that few papers have been published on this subject.

In the present study, we examined use of the GP’s waiting room as a vector for the dissemination of health information. Most waiting rooms have a number of posters on display. Posters are widely used for health promotion because they constitute an inexpensive way of providing written information to a large proportion of the population [5]. This educational approach can be used to encourage people to implement preventive measures, undergo screening or adopt good treatment practices. Despite the limitations of posters as a means of health education, previous studies have reported that poster displays in hospitals, waiting rooms and emergency departments are an effective vehicle for health education on several topics (such as antismoking campaigns, family planning, AIDS prevention and promotion of physical activity) [6-11].

We therefore tried to evaluate French GPs’ opinions on their waiting rooms as a vector for health promotion. The study’s primary objective was to describe how GPs made use of the waiting room and how they chose the media displayed. The study’s secondary objective was to describe the health promotion documents present in waiting rooms.

## Methods

A semi-structured, face-to-face interview was used to study a representative sample of GPs. This approach was combined with analysis of the health information documents displayed or distributed in waiting rooms.

The study population was selected in order to be representative of GPs in the Picardy region of northern France. In order to interview physicians of all ages and different working environments, and with the human resources at our disposal, we arbitrarily decided to meet 60 physicians. Based on an expected participation rate of 30%, 200 GPs in the region were contacted by sending an invitation by registered mail. The sample was generated by stratified randomisation according to gender, age and type of practice (rural, semi-rural or urban). A telephone reminder was required to obtain the desired number of participants. The first 60 GPs agreeing to participate were included.

The study questionnaire (Additional file 1) consisted of 20 items addressed in a variable order, depending on the course of the interview. A survey design was applied with a mixed-methods approach including open-ended and multiple-choice questions. After providing sociodemographic information, doctors were asked to freely comment on their involvement in health education, their perception of the function of the waiting room, their opinion on health information provided by documents and their patients’ responsiveness to health information documents. The investigator initiated discussion of certain topics if the GP did not spontaneously volunteer an answer.

The semi-structured interviews were all performed over a period of three consecutive months by the same investigator, who also used a structured, analytical grid to assess the number, type, source and subject of the documents available in the waiting room. This tool was designed from the literature review and the defined objectives of the study and allowed systematic interview of the physicians.

The physicians’ responses were transcribed and analysed qualitatively to identify the concepts developed during the interview. The transcripts were entered into Tropes software for content analysis and coded according to the session headings [12]. Tropes software is designed for semantic classification, keyword extraction, and linguistic and qualitative analysis [13]. After analysis of the content, recoding of the discussions was performed based on the themes that emerged from the analysis. The responses were then grouped into categories in order to summarize the GPs’ opinions and actions.

A descriptive analysis of quantitative data was performed on SPSS software (version 11.0, SPSS Inc., Chicago, IL, USA).

Ethics committee approval is not required for studies with no impact on physician practice or patient care.

## Results

### Description of the sample

Sixty GPs participated in the study. They were predominantly male (80%) with practices fairly evenly distributed between rural, semi-rural and urban areas (30%, 33% and 37%, respectively).

One third of physicians were under the age of 45, 57% were aged between 45 and 59 and 10% were aged 60 or over. Forty-eight percent of GPs had been in practice for over 20 years, 30% had been in practice for between 10 and 20 years and 22% had been in practice for less than 10 years. The physicians who participated were representative of the GP population (Table 1).

**Table 1:** Distribution of the GP’s population and the GP sample of the study

**Gp population**					**Sample**	
**Sex**	**Age**	**Practice environment**	**N**	**%**	**Expected (N)**	**Included (N)**
Male	30-44	rural	26	5.2	3	3
		semi-rural	32	6.4	4	4
		urban	39	7.8	5	5
	45-59	rural	75	15	9	9
		semi-rural	84	16.8	10	10
		urban	90	18	11	11
	≥60	rural	13	2.6	2	2
		semi-rural	17	3.4	2	2
		urban	17	3.4	2	2
Female	30-44	rural	24	4.8	3	3
		semi-rural	17	3.4	2	2
		urban	28	2.2	1	1
	45-59	rural	11	2.2	1	1
		semi-urban	17	3.4	2	2
		urban	8	1.6	1	2
	≥60	rural	1	0.2	0	1
		semi-urban	2	0.4	0	0
		urban	0	0	0	0

### Health information

Most GPs considered that the patient demand for health information had increased over recent years. The majority of GPs considered that health information was their responsibility. They also mentioned the role of institutional stakeholders, such as national prevention and health education agencies, national health insurance, the French Ministry of Health and regional healthcare agencies. The mainstream broadcast media and printed media were also cited. More anecdotally, physicians also mentioned pharmacists and teachers as sometimes being involved in the provision of health information.

General practitioners considered that television was the best medium for disseminating health information, followed by information brochures, printed media and poster campaigns. Internet ranked only fifth - just ahead of radio, the medium mentioned least often by the GPs.

All physicians agreed that a waiting room must be pleasant, relaxing and enjoyable. It must enable patients waiting for their consultation to “forget their disease” and reduce any anxiety that may be involved.

Twenty-five of the 60 GPs spontaneously indicated that the waiting room could play a role in patient information. The great majority of the other 35 GPs agreed on this role of the waiting room, when the subject was raised. Only 6 physicians expressed scepticism concerning this role.

The physicians regularly received patient-targeted health information posters or brochures on a daily basis (n = 7), several times a week (n = 5), one to three times a month (n = 38) or less than once a month (n = 10).

Most physicians who received posters and brochures for patients read them carefully. They generally considered that these documents were well tailored for their patients. It is noteworthy that the GPs’ views on a document’s relevance determines the subsequent distribution or display of the document. A minority of physicians distributed material after reading it quickly (n = 10), whereas 14 always threw the material away without reading it at all (n = 14).

Physicians stated that they distributed health information materials in order to improve their relationships with their patients. The reasons most frequently cited were to encourage screening, provide health education information, more easily address a delicate subject, facilitate dialogue with patients and facilitate the prescription of certain treatments.

Some physicians decided not to distribute documents which they thought would raise their patients’ anxiety levels. Others considered that health information campaigns were inadequate, unclear or of no interest to their patients.

Renewal of information documents was frequently mentioned. The great majority of GPs said that the documents displayed had been received during the previous three months and nearly one half stated that the documents had been received during the previous month.

Most physicians did not take the initiative to obtain health information documents. Only four physicians stated that they regularly ordered documents, while others ordered documents occasionally (n = 18). GPs contacted various drug companies, the French National Institute for Prevention and Health Education and national health insurance to obtain health information documents.

The great majority of physicians felt that patients consulted the brochures made available in their waiting rooms and found them helpful. More than two thirds of physicians stated that patients had (very occasionally) discussed prevention with them after reading a document placed in the waiting room.

Audiovisual aids were not widely used. Only one GP used this vector to broadcast health prevention or education messages. Other physicians stated that they were against the use of audiovisual aids (primarily because the necessary equipment was not available in their rooms).

### Analysis of waiting room documents

Posters were displayed in 47 waiting rooms (78%) with an average of 6.9 posters per waiting room. The posters mainly consisted of health information posters, but also featured information on organizational aspects of the GP’s practice (opening hours, the presence of a medical student during the consultation, fees, emergency telephone numbers, etc.).

The health topics mostly frequently addressed by the posters were vaccination (n = 17 waiting rooms), nutrition (n = 16) and emergency telephone numbers (n = 11). Waiting room posters less frequently concerned smoking (n = 6) and alcohol (n = 6). Other topics are listed in Table 2. Only 8 physicians had chosen a single theme for all posters in their waiting rooms.

**Table 2:** Health topics most commonly addressed in waiting room documents, decreasing order of frequency in the waiting room

**Topics on posters**	**Number of the waiting rooms**	**Topics in brochures**	**Number of waiting rooms**
1. vaccination	17	1. nutrition	19
2. nutrition	16	2. cardiovascular disease	17
3. emergency phone numbers	11	3. vaccination	16
4. drugs of abuse	9	4. information on technical aids for patients at home	15
5. cardiovascular disease	9	5. a cancer information hotline	15
6. information medical consultations at home	8	6. asthma	11
7. tobacco	6	7. skin cancer	11
8. asthma	6	8. screening for cervical cancer	10
9. abortion	6	9. osteoporosis	10
10. violence against women	6	10. emergency phone number	9
11. alcohol	6	11. bronchiolitis	9
12. hepatitis C	5	12. herpes	8
13. blood or organ donation	5	contraceptive	8

Most of the posters displayed in waiting rooms (n = 32) were derived from institutional sources (the French National Institute for Prevention and Health Education, national health insurance, ministries and local authorities), followed by patient associations and medical associations (n = 20) and drug companies (n = 18). In some cases, the non-profit organisations supplying posters were linked to institutional bodies (n = 11) or drug companies (n = 11).

Brochures were available in 34 waiting rooms (57%) with an average of 10.7 per waiting room.

The topics most frequently addressed in brochures were nutrition (in 19 waiting rooms), cardiovascular disease (n = 17) and vaccines (n = 16). Other topics are listed in Table 1. Only two physicians had chosen a single theme for all brochures in their waiting room.

Other information documents were present in the physicians’ waiting rooms. General magazines were present in almost all waiting rooms (58 out of 60), with an average of 22 per waiting room. Magazines specialized in health and well-being were available in only six waiting rooms. Similarly, children’s magazines were rarely available (n = 13) and only two children’s magazines had an educational content.

## Discussion

### Health information documents and communication strategy

The GPs interviewed agreed on the growing interest of their patients in health information and the physician’s role in this area. GPs considered that the waiting room can play a role in health education of patients and distribution of documents during the waiting time. However, none of the physicians appeared to have integrated these documents into a health communication strategy. Very few physicians distributed documents focused on a single theme. Similarly, very few GPs ordered the documents that they wished to distribute. Physicians generally had a passive attitude; they simply distributed documents that they considered to be relevant with no clear communication strategies concerning high-priority health themes.

Although posters can increase awareness of health promotion issues, [6] their messages are not necessarily effective in changing patients’ behaviour and lifestyles. Ashe et al. showed that public education in the form of waiting room posters was not sufficient to decrease antibiotic prescriptions [14].

However, other authors have shown that it may be relevant to make waiting room media part of an active health education strategy [15]. Despite the known limitations of posters as a means of health promotion (especially when used alone), [16] their use in general practice may still be justified. We believe that posters should be integrated into the physician’s own education strategy. A link between posters and/or brochures distributed in the waiting room and preventive actions conducted by the physician during the consultation could reinforce these messages. These coordinated campaigns between waiting room messages and preventive actions should ideally be limited in time in order to mobilize the patients’ attention. This type of coordinated campaign needs to be further evaluated. We recommend that the posters and/or brochures distributed in such a campaign should preferably be devoted to a single theme, or at least consistent themes.

This strategy can echo large-scale, national programmes or may be customized according to the priorities identified by the physician. The message delivered by the posters should be reinforced during consultations; for example, the patient could be given a brochure providing additional information.

In some medical specialties, videos have been successfully used to help patients understand what to expect during their care. In particular, patient education videos can be a relatively effective means of describing healthcare procedures. This can facilitate compliance with care [17,18]. This medium can increase the patient’s understanding of and satisfaction with their care, [19-22] and decrease their anxiety levels. Although videos can effectively improve knowledge about a complex topic, [20] or increase the participation in a prevention programme, [23] their use in waiting rooms is subject to technical constraints [24]. Information videos are more expensive to produce and broadcast in waiting rooms, which constitutes an obstacle to their development in a context of limited resources [22,25]. Some structural barriers, such as shared waiting areas, require innovative alternatives [26].

### Information and anxiety

GPs expressed the concern that waiting room documents could induce or accentuate the patient’s anxiety. The waiting time itself can be stressful. Studies in hospital settings have shown that patients should be offered alternative activities during their waiting time. For example, patients suggest that meetings with other healthcare professionals (psychologists, nurses, dieticians, etc.) could be organized while waiting to consult a physician. Some authors have even suggested entertainment or leisure activities (music therapy, drawing courses, library services, TV, etc.) [27]. A number of studies have assessed the value of various techniques designed to reduce patient anxiety in the waiting room [28].

However, the provision of waiting room information may reduce anxiety when it concerns a care procedure that the patient will subsequently use or experience [19].

### Sources of information documents

Whereas most waiting room information was derived from official bodies (the French National Institute for Prevention and Health Education, national health insurance, etc.), non-profit associations and drug companies also distributed many documents. Physicians must carefully assess the relevance of the topics addressed and their rigorous scientific content. Physicians are free to choose their health information priorities in their own practice. However, GPs are the effectors of public health policy and distribution of documents from drug companies and associations may raise issues of scientific credibility and conflicts of interest [29].

Very few physicians ordered their waiting room documents and most adopted a passive attitude in relation to distribution of these documents. Fortunately, most doctors read the documents before distributing them, allowing them to at least critically review the document’s format, publisher and the quality of its content.

### Study limitations

The descriptive nature of this study does not allow any formal conclusions to be drawn. However, our results contribute to the understanding of how information materials are used in the GP’s waiting room.

The study population was intended to be representative of the GPs in our region. However, the 60 doctors who agreed to be interviewed were probably more interested in this subject, which constitutes a source of bias.

Further studies are therefore required to extend the results of the present study and to assess the impact of a structured communication strategy on patient information and the doctor-patient relationship.

## Conclusion

Health information is an issue for individual patients, healthcare professionals and health authorities. Physicians use their waiting room to disseminate a broad range of information documents. However, this diffusion of information appears to be uncoordinated and lacks a defined communication strategy.

We believe that it might be of value to match the topics of the documents available in the waiting room to the prevention practices introduced during the consultation. Information brochures could be given to patients during the consultation in order to reinforce the healthcare professional’s educational message. Effective coordination between the national agencies that distribute information material and doctors could enable public health programmes and GPs to deliver a more coherent message. The health education messages relayed by GPs in waiting rooms and consultations may have greater weight if they are part of national, mass-media campaigns.

## Competing interests

The authors declare that they have no competing interests.

## Authors’ contributions

MG performed analysis and drafted the manuscript. HI designed the study, and participated in its design and coordination. CM helped to draft the manuscript OG designed the study, and participated in its design and coordination and helped to draft the manuscript. All authors read and approved the final manuscript.

## Supplementary Material

Additional file 1 **Appendix 1.** Study questionnaire. Click here for file
