# Supplementary material for: The waiting room: vector for health education? the general practitioner’s point of view
Source: BMC Res Notes. 2012 Sep 18;5:511. doi: 10.1186/1756-0500-5-511 (PMC3599884; doi:10.1186/1756-0500-5-511)
Supplement: Additional file 1 — Appendix 1. Study questionnaire. [file 1756-0500-5-511-S1.pdf]

Appendix 1: Study questionnaire

A. Questions for GPs.

1. Gender: Male female

2. Age: _ _ _ _ _ _

3. For how long have you been a GP? _ _ _ _ _ _

4. Practice environment

 Urban

 Semi-rural

 Rural

5. Who do you believe is responsible for health information?

_ _

_ _

_ _

_ _

_ _

_ _

_ _

_ _

6. How would you assess the growing demand for health information over the last 10 years?

_ _

_ _

_ _

_ _

_ _

_ _

_ _

_ _

7. What do you think are the most appropriate media to disseminate health information?

(TV, Radio, Posters, brochures, newspapers, Internet, etc.)

_ _

_ _

_ _

_ _

_ _

_ _

_ _

_ _

8. What functions do you assign to your waiting room?

_ _

_ _

_ _

_ _

_ _

_ _

_ _

_ _

_ _

9. How often do you receive posters and/or health information brochures?

☐ Daily

☐ Several times a week

☐ More than once a month

☐ Once a month

☐ Less than once a month

10. Overall, do you consider these posters and/or brochures to be adapted to the patient?

☐ Yes

☐ Rather yes

☐ Rather not

☐ No, not at all

☐ Neither yes nor no

11. What do you do when you receive posters and/or brochures?

☐ You throw them away.

☐ You distribute them after having read them quickly.

☐ You read them carefully an only distribute those that you consider to be relevant.

☐ Other: ____

____

12. If you distribute them, why?

____

____

____

____

____

____

____

____

13.If you do not distribute them, why?

____

____

____

____

____

____

_ _

_ _

14. Have you ever specifically ordered posters and/or brochures?

- ☐ Never
- ☐ Rarely
- ☐ Sometimes
- ☐ Often

15. If you have ordered posters and/or brochures, from which institution? _ _ _ _ _ _ _ _ _

_ _

_ _

16. Would you say that patients use the brochures made available to them?

17. Do patients talk with you about prevention issues after reading brochures in your waiting room?

☐ Yes ☐ No

17.1. If yes, would you say this happens:

☐ Very occasionally

☐ Sometimes

☐ Often

18. Do you distribute educational video messages in your waiting room?

☐ Yes ☐ No

19. What do you think of the dissemination of audiovisual messages for health education?

_ _

_ _

_ _

_ _

_ _

_ _

_ _

_ _

B. Waiting room documents

1) Posters

Number: _ _ _ _ _ _ _ _ _ _ _ _ _

Themes: _

_ _

_ _

_ _

Source: _

_ _

_ _

_ _

2) Brochures

Number: _ _ _ _ _ _ _ _ _ _ _ _ _

Themes: _

_ _

_ _

_ _

Source: _

_ _

_ _

_ _
